# The power of peer networking for improving STEM faculty job applications: a successful pilot programme

**DOI:** 10.1098/rspb.2023.0124

**Published:** 2023-04-26

**Authors:** Carlos M. Guardia, Erin Kane, Alison G. Tebo, Anna A. W. M. Sanders, Devrim Kaya, Kathleen E. Grogan

**Affiliations:** ^1^ Neurosciences and Cellular and Structural Biology Division, Eunice Kennedy Shriver National Institute of Child Health and Human Development, National Institutes of Health, Bethesda, MD, USA; ^2^ Reproductive and Developmental Biology Laboratory, National Institute of Environmental Health Sciences, National Institutes of Health, Research Triangle Park, Durham, NC, USA; ^3^ Department of Medicine, Chobanian & Avedisian School of Medicine, Boston University, Boston, MA, USA; ^4^ Howard Hughes Medical Institute—Janelia Research Campus, Ashburn, VA, USA; ^5^ Department of Biochemistry and Molecular Biology, Roy J. and Lucille A. Carver College of Medicine, University of Iowa, Iowa City, IA, USA; ^6^ School of Chemical, Biological, and Environmental Engineering, Oregon State University, Corvallis, OR, USA; ^7^ Departments of Anthropology and Biological Sciences, University of Cincinnati, Cincinnati, OH, USA

**Keywords:** peer-reviewing, faculty job applications, mentoring, STEM, postdoc

## Abstract

To attain a faculty position, postdoctoral fellows submit job applications that require considerable time and effort to produce. Although mentors and colleagues review these applications, postdocs rarely receive iterative feedback from reviewers with the breadth of expertise typically found on an academic search committee. To address this gap, we describe an international peer-reviewing programme for postdocs across disciplines to receive reciprocal, iterative feedback on faculty applications. A participant survey revealed that nearly all participants would recommend the programme to others. Furthermore, our programme was more likely to attract postdocs who struggled to find mentoring, possibly because of their identity as a woman or member of an underrepresented population in STEM or because they changed fields. Between 2018 and 2021, our programme provided nearly 150 early career academics with a diverse and supportive community of peer mentors during the difficult search for a faculty position and continues to do so today. As the transition from postdoc to faculty represents the largest ‘leak’ in the academic pipeline, implementation of similar programmes by universities or professional societies would provide psycho-social support necessary to prevent attrition of individuals from underrepresented populations as well as increase the chances of success for early career academics in their search for independence.

## 1. Introduction

The purpose of a postdoctoral appointment is the acquisition of additional skills or training post-PhD in preparation for transitioning into an independent position as a primary scientific investigator [1], originally in academia [2], although the function has broadened to include training for a multitude of other career paths [3]. Thus, by definition, postdoctoral appointments are temporary (average = approx. 2.7 years [3–5] but see [6]) and individuals may complete multiple postdoctoral positions in different laboratories before gaining independence [7–9]. Because of the limited duration and lack of funding security for postdoctoral positions as well as the highly competitive nature of the tenure-track faculty job market [5,10–13], postdocs spend a significant amount of time searching for their next appointment. Some will begin to search for the next position as soon as they begin their current position, and many apply for postdoctoral and tenure-track positions simultaneously over multiple years [6].

This transition from postdoctoral scholar to tenure-track faculty is a pivotal moment for early career researchers, as it represents the most likely time to ‘leak’ out of academia. Nearly 60% of biomedical or life sciences graduate students transition to postdoctoral appointments but less than 20% of these PhDs attain a tenure-track faculty appointment, less than half the number that report a faculty job as their top choice [1]. This ‘leak’ is not automatically a negative outcome as nearly all these individuals successfully transition to industry or government [1]. However, the transition from postdoctoral scholar to faculty also represents the biggest barrier to equitable representation for women and people from marginalized groups in higher education. For example, in the United States, women and non-white individuals of all racial or ethnic backgrounds are significantly less likely to transition from postdoc to faculty. In fact, research suggests that individuals who identify as Black or African American, American Indian or Alaska Native, Hispanic or Latinx, or Native Hawaiian or Pacific Islanders are 60% less likely to transition from graduate student to a faculty position compared to white individuals [14–18]. Structural and systemic inequities and barriers notwithstanding, improving opportunities for senior and peer mentorship and support could reduce this failure to retain talented early career researchers, for example, by providing opportunities during the faculty application process.

The specific documents requested in an application packet for a faculty position (faculty applications) differ between institutions, especially between geographical locations (e.g. North America versus Europe versus Asia), but generally consist of multiple highly crafted documents. In the USA, the packet usually includes a curriculum vitae (CV), cover letter and statements of research, teaching, and, sometimes, diversity (for a description of these documents and the faculty application process, see [6,19]). To maximize the chance of success, applicants spend a significant amount of time writing and polishing these documents. Numerous opinion and advice pieces have been published on how to craft these documents (e.g. [20–23]). In addition, institutional Offices of Postdoctoral Education, the National Postdoctoral Association in the USA, and some national and international scientific societies host seminars/webinars and publish guidelines that provide extensive advice on the structure and content of these documents (e.g. [8,24]). Almost all these seminars and advice columns direct postdocs to solicit feedback from a wide circle of peers and mentors. However, non-white, LGBTQ+ and/or female individuals are more likely to lack access to the extensive circle of peers and mentors recommended [25–31]. As a result, these postdocs will struggle to receive the same level of feedback as those with large, supportive academic networks and may, therefore, struggle to bring their application materials to a competitive level.

Formal and informal mentors are nearly always willing to provide constructive feedback [32]; however, some mentors may be unwilling to spend time on this task or are unable to offer useful feedback, especially for postdocs applying for positions in fields, institutions or region-specific systems that differ from the mentors' own institutions or institutional experiences [10,32–34]. Postdoctoral peers and senior graduate students can serve as additional reviewers, but most research groups only have a couple of postdocs at a time or may have none [4,35]. Moreover, the breadth of scientific expertise represented within a research group or postdoc's network rarely matches the breadth of expertise represented by search committees in academia. Thus, while one's colleagues/labmates may be able to comment on the structure and the science within a job application, they may not be able to assess if a research statement is broad and general enough to appeal to the wider audience represented by a search committee, nor whether the application matches the expected format for institution type and location. Furthermore, while mentors and peers may be happy to review a document a few times, most mentors and peers lack the time to provide multiple rounds of feedback on greater than 10 pages of job application materials.

Postdocs could benefit from a variety of options for getting feedback on their job application materials, but opportunities to have job applications critiqued repeatedly by a broad scientific audience are generally scarce. This scarcity is magnified for postdocs employed at under-resourced or isolated institutions, that may not have sufficient support systems for early career researchers and postdocs from marginalized groups, who are less likely to have a large peer network and access to mentoring and support [25,31]. Here, we present a potential solution in the form of an open and inclusive international peer review programme for job application materials that has been running since 2018. In this Programme, participants have the opportunity to repeatedly share all or part of their application package with a small group of peer reviewers. They engage in reciprocal, constructive commentary in a supportive and encouraging manner with the ultimate desire of seeing each other succeed in attaining an independent faculty position.

Peer review programmes are most widely used for manuscript ([36–38], but see [39,40]) and grant application reviews ([41–43] but see [44,45]), but have been successfully implemented in many other contexts for purposes of professional development and community building, especially for postdocs and early career researchers [46–49]. Our Programme is designed to provide opportunities for postdocs and other early career researchers to give and receive substantive feedback on faculty applications as well as provide significant peer support and opportunities for networking and mentoring throughout the job application process. Together, increasing opportunities for professional development, community building and networking are essential to improving the retention of women and underrepresented minorities and increasing diversity and inclusion in STEM and academia [27–29,50,51].

This paper has three goals: (i) to describe the focus and organizational details of our Programme; (ii) to use survey data to assess the experiences of participants and non-participants on the benefits and limitations of this type of programme; and (iii) to suggest methods of implementation for other organizations (i.e. Offices of Postdoctoral Education, postdoctoral societies or scientific societies). By showcasing how our Programme improved feelings of self-confidence and well-being through peer support and community building during the ‘leakiest’ transition point in academia, we suggest that similar programmes could be critical to retaining a diverse early career academic workforce.

## 2. Programme description

**Figure 1.**
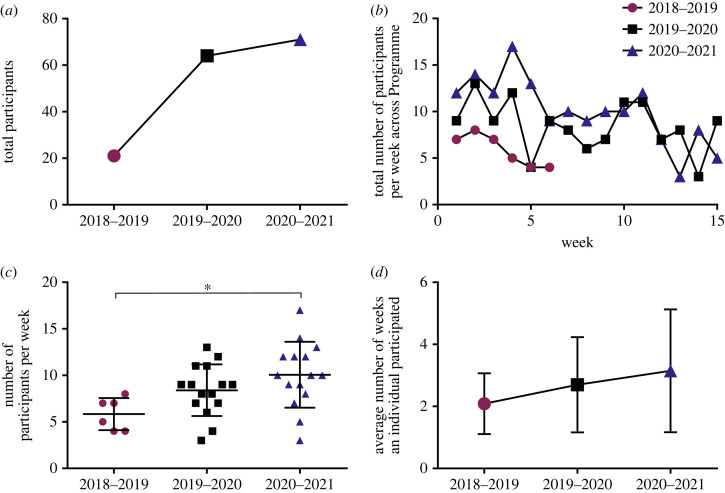
Programme participation for the FuturePI Reviewing Groups Programme from 2018 to 2021. (*a*) The total number of unique individuals who participated in the reviewing Programme per year, (*b*) the total number and (*c*) average of individuals who participated each week of the Programme by year, and (*d*) the average number of weeks an individual participated by year.

This Programme began organically after its founder, senior author Dr Grogan, observed frequent requests for peer review of job application materials on the FuturePI (Principal Investigator) Slack Group (https://futurepislack.wordpress.com/) and realized the group could benefit from a programme for formal peer-reviewing of application materials during the job application season. The FuturePI Slack community is a space for postdoctoral researchers, primarily although not exclusively from the biomedical fields who are interested in or planning a faculty career, to engage with and learn from one another. In its first year, the 2018 FuturePI Reviewing Groups Programme (hereafter called the Programme) ran for seven weeks, from mid-August to mid-October. Beginning in 2019, the timeline expanded to 15 weeks, from early August to the end of November (see electronic supplementary material). The Programme is announced through the FuturePI Slack #general and #Jobapp_reviewer channels two weeks before its start to give participants time to sign up on an open-access Google Sheet. Participants are asked to provide their names, email addresses, general field of study, the type of jobs they are applying to and to indicate which weeks they would like to participate (see electronic supplementary material, figure S4 for example). Reviewing groups are organized weekly, with sign-ups for the upcoming week closing on Sunday morning. All interested participants for a given week are emailed the day before groups are assigned to confirm their willingness to participate that week, and then reviewing groups are organized the next morning. Groups are organized primarily by field and type of job that participants are applying to—for example, one group might consist of neuroscience candidates applying to R1 (Carnegie classification of ‘Very High Research activity’) positions while another group might consist of more general biology candidates primarily applying for R2 (Carnegie classification of ‘High Research activity’), PUI (primarily undergraduate institution) or small liberal arts college (SLAC) positions. At the start of each week, individual reviewing groups are emailed contact information for their group and instructed to send whatever documents they want to be reviewed that day and asked to provide feedback on each other's documents by the end of the week (for example announcement, confirmation and assignment emails; see electronic supplementary material). To illustrate the scheduling, during the first three years of operation, organizers confirmed participation interest on Sunday, assigned and emailed groups on Monday and participants were asked to provide feedback for group members' documents by Friday, but this schedule can be adjusted easily. Participation in the Programme is open to any current member of FuturePI Slack and the Programme has grown since its first year, from 21 unique participants in 2018 to 71 in 2020 and 69 in 2021 (figure 1*a*). The number of unique individuals who participate each week varies considerably (mean = 8.7, range = 3–17; figure 1*b*), but has steadily increased since the Programme's inception (*F* = 4.363, *p* = 0.02; figure 1*c*). Additionally, the number of times that any given individual participates has also increased, although not significantly (*F* = 1.039, *p* = 0.36; figure 1*d*).

## 3. Feedback survey for Programme participants and non-participants

Given the increase in participation and anecdotal commentary about the benefits of participating in the Programme, we developed a survey for participants and non-participants in the Programme to assess participants' experiences and identify areas for improvement (electronic supplementary material, table S1). Participants were surveyed to document who participates in the Programme, why they do so, and what aspects of the Programme were helpful or could be improved. We surveyed non-participants to document any differences in demographics between participants and non-participants, as well as to ask why individuals choose not to participate and if we could make improvements that might encourage non-participants to participate in the future. All individuals who participated in the survey were members of the FuturePI Slack community and are hereafter referred to as respondents, regardless of whether they participated in the Programme or not. Respondents who participated in the Programme are referred to as participants (59.8% of respondents) and those who did not participate as non-participants (40.2% of respondents). We collected responses to this survey during the spring of 2021 (University of Cincinnati IRB no. 2020-0891). To maintain anonymity and promote ease of response, the survey, which took approximately 5–10 min to complete, was conducted through Google Forms and all questions were optional, except the IRB permission. Respondents were recruited via messages posted on various FuturePI Slack channels as well as through direct emails to previous and current participants in the Programme. Responses were collected from early February to the end of April 2021. Data were organized and analysed in Excel, OriginPro2015 and GraphPad Prism 6. Answers to open-ended questions were stripped of white space, punctuation, numbers and English ‘stop words’ (e.g. and, the, is) using the package *tm* (v.0.7-8) [52] and were manually edited to change plural nouns to singular nouns and stem words with the same root (e.g. standardizing review, reviews and reviewed as ‘review’). Then, word clouds were generated using the package *wordcloud* (v.2.6) [53] in Rstudio.

### (a) Notes on the use of terminology for sex/gender and race/ethnicity

Reporting on sex/gender and race/ethnicity data can involve terminology that changes over time as more open discussion of variation in identities allows for improved societal understanding and acceptance. The original survey asked the question ‘How would you define your gender?’ and included options for male, female, non-binary and prefer not to say. These options were used to increase our ability to compare our results to previous surveys which used the same terminology. However, we want to note that male and female are generally used to discuss biological sex while man and woman are used to discuss gender identity. Although we use female throughout this paper to match the options offered in the survey, were we to conduct this survey today, we would use the options of man, woman, non-binary and prefer not to say, and would likely include a question about sexual orientation in order to better capture information about LGBTQ+ individuals in STEM. Additionally, although we included an option to describe one's ethnicity or race in that question, we may use different terminology in the future. For example, when asked to describe one's ethnicity or race, options included American Indian or Alaska Native, Asian, Black or African American, Native Hawaiian or Pacific Islander, Latinx or Hispanic, White and Other. Currently, there is debate about the usage of Latinx versus Hispanic. We acknowledge that both terms are controversial for different reasons, but we chose to use both terms to be more inclusive and to reflect the options offered to participants in our survey and the comparative US Postdoc Survey [3].

### (b) Survey respondent demographics

**Figure 2.**
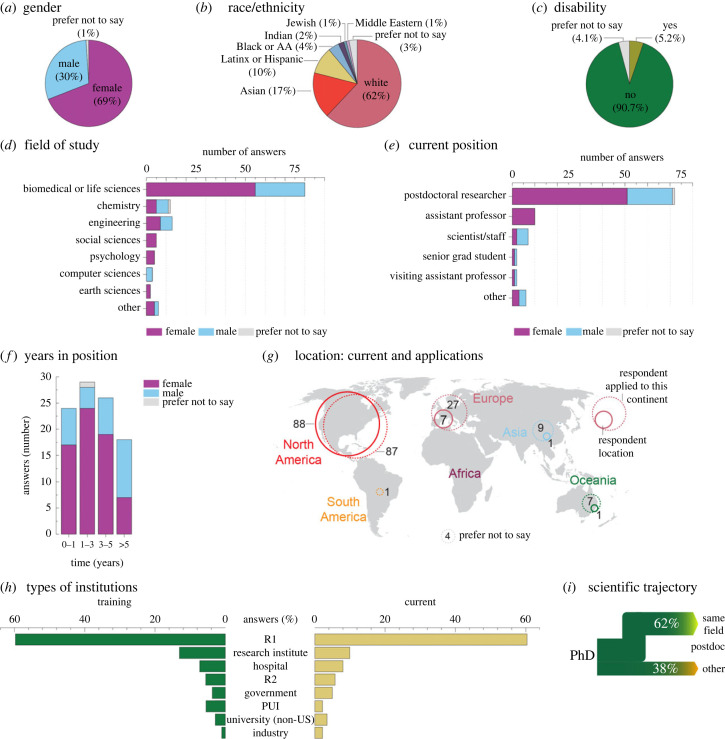
Self-identified demographics and career information of survey respondents, which included both Programme participants and non-participants: the distribution of survey respondents by (*a*) gender, (*b*) race/ethnicity and (*c*) disability status; the distribution of respondents, split by gender, according to (*d*) field of study, (*e*) current position title and (*f*) years in current position; (*g*) map of the current location of survey respondents (solid circles) compared to the location of positions to which respondents applied (dashed circles); (*h*) the type of institution at which survey respondents were or are employed; (*i*) the representation of percentage of survey respondents who either remained in the same field as their PhD training or switched scientific fields post-graduation.

Overall, we found that both our survey and Programme recruited more women and more individuals who identified as having a disability than expected, but percentages of respondent race/ethnicity matched expectations based on the general US postdoctoral population. The majority of our survey respondents (*n* = 97), which included both Programme participants and non-participants, identified as female (69%), white (62%), and did not identify as disabled (90.7%; figure 2*a–c*). The respondents of this study were significantly more likely to identify as female compared to the respondents of the 2016 USA National Postdoctoral Survey and a recent survey of faculty job applicants, in which 53% and 48.2% of respondents identified as female, respectively (*χ*^2^ = 12.97, *p* = 0.0015) [3,6] (but see [54]). Given differences in category labels, we were only able to statistically compare the self-identified race/ethnicity of the respondents for white, Black or African American and Latinx/Hispanic identities. Our survey included significantly more African American or Black respondents compared to the US postdoctoral population (*χ*^2^ = 26.00, *p* < 0.0001) [3]. Over 5% of our respondents identified as having a disability, more than the 0.6% reported in the USA National Postdoc Survey but similar to the 6% (12 out of 175) reported by a similar survey of postdocs and early career researchers in ecology and evolution [3,55].

Compared to a recent survey of faculty applicants (also organized in part through FuturePI Slack) [6], the respondent population of this study was similarly biased towards postdocs over graduate students, staff scientists, or assistant professors and had many more respondents working in biomedical or life sciences, with less than 15 respondents each from chemistry, engineering, social sciences and other fields (figure 2*d*,*e*), which aligns with the primary focus of FuturePI Slack towards the biomedical and life sciences. Survey respondents were approximately evenly distributed across the lengths of job tenure from 0 to 5 years (figure 2*f*). Although the population skews towards individuals in the USA, FuturePI Slack is an international organization with the survey respondents located across the globe and applying to a similarly broad geographical range of jobs (figure 2*g*), although the majority were located in and/or applying to jobs in North America. Nearly 54% of respondents were currently employed at an R1 institution, with the rest of the respondents employed at research institutes, hospitals, R2 or PUI institutions, or in government positions (figure 2*h*). Fewer respondents (40.8%) reported being trained at an R1 institution compared to other types of institutions. Interestingly, nearly 40% of our survey respondents reported they had changed fields between their PhD and current position and this finding did not differ by gender (*χ*^2^ = 0.003, *p* = 0.95) or race (*χ*^2^ = 1.61, *p* = 0.2; figure 2*i*). Of the 97 survey respondents, 58 were participants in the Programme at least once in the previous three years while 39 respondents were non-participants.

### (c) Job searches of survey respondents

**Figure 3.**
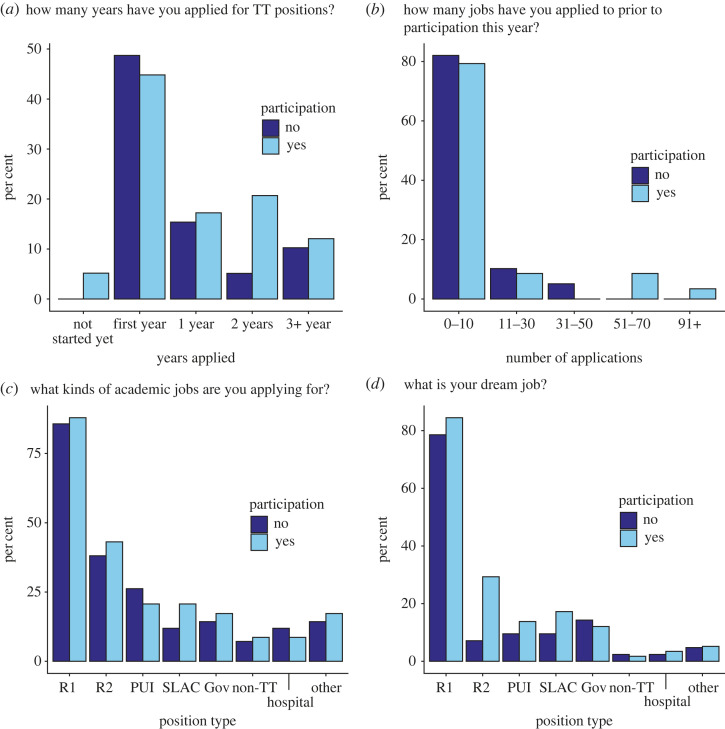
Comparison of the faculty job application journey for participants and non-participants: (*a*) the number of years spent searching for a faculty job, (*b*) number of job applications and (*c*) types of job applications submitted by participants and non-participants, and (*d*) the dream job for participants and non-participants. For (*c*,*d*), other includes applications for faculty, research scientist and postdoctoral positions at professional schools, research institutes, non-profits and industry companies.

In assessing job application trajectory differences between participants and non-participants, we found that although many respondents were in the first year of their job search, more participants had been on the job market for at least 3 years compared to non-participants and participants had submitted higher numbers of applications. Respondents in their first year of the job market were the largest portion (greater than 40% of total) for both participants and non-participants (figure 3*a*). However, more than 30% of participants were in their third or greater year of the job search compared with only approximately 15% of non-participants although this difference was not statistically significant (*χ*^2^ = 8.94, *p* = 0.06; figure 3*a*, left two columns). Notably, three participants were in their sixth year on the job market. The majority of respondents (approx. 80%) had submitted ten or fewer applications and we found no overall significant differences between the number of applications submitted between participants compared to non-participants (figure 3*b*). Remarkably, however, some of the participants had already submitted substantial numbers of applications, including six individuals who reported submitting greater than 90 applications. In comparison, none of the non-participants had submitted more than 50 applications at the time of the survey (figure 3*b*). From these data, we observed that participants tended to be further along in their job search trajectory, having spent more years searching for jobs and submitted many more applications, although these trends were not statistically significant.

We found that while both participants and non-participants were applying to R1 positions and considered this type of institution their dream, participants were more likely to apply to a wider range of position types (*χ*^2^ = 1.86, *p* = 0.96; figure 3*c*) and to report that their dream job also included other types of institutions such as R2 and PUIs (*χ*^2^ = 9.68, *p* = 0.21; figure 3*d*). However, these trends were not statistically significant. Specifically, among all respondents, R1 universities were the most applied to position (*n* = 51 participants and 33 non-participants of 97 total respondents; figure 3*c*) and most frequently reported dream position (*n* = 49 participants and 31 non-participants), with approximately 80% of respondents reporting their dream position would be at an R1 institution (figure 3*d*). Participants applied to more non-R1 positions compared to non-participants, which could be due to the career stage and experience of the participant in previous job cycles leading to a broadening of their targeted institution. More participants also reported that their dream jobs were at non-R1 educational institutions, e.g. R2 universities, PUIs and SLACs.

### (d) Mentorship and peer support of survey respondents

**Figure 4.**
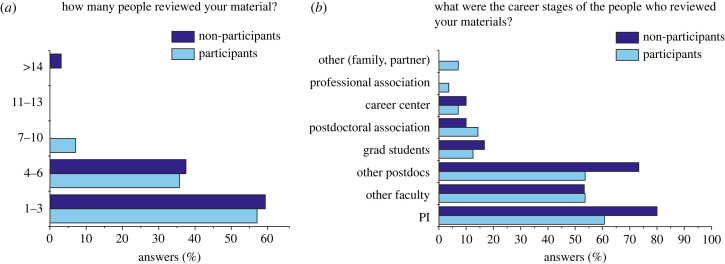
Information on the availability of reviewers for survey respondents' job application materials: (*a*) the number and (*b*) career stages of reviewers recruited by survey respondents to review their job application materials.

Regarding the mentorship and peer support available for respondents, we found that although participants and non-participants generally had the same number of reviewers for their job application materials (figure 4*a*), participants were less likely to ask faculty supervisors or postdoctoral colleagues to review their materials (figure 4*b*). Most of the respondents had their application materials reviewed by 1–3 people (55–60%) or 4–6 people (35–40%) outside the Programme (figure 4*a*). These reviewers consisted of mostly PIs, other faculty and postdoctoral colleagues, but the percentage differed between participants and non-participants (figure 4*b*). For participants, slightly over 60% had their PI review their materials, approximately 53% had other faculty reviewers, and nearly 55% recruited other postdoctoral colleagues to review their materials. By contrast, for non-participants, approximately 80% were able to have their PI review their materials, approximately 53% had other faculty reviewers, and nearly 75% were able to recruit other postdoctoral colleagues to review their materials. In other words, participants were less likely to have their job application materials reviewed by their PI or other postdoctoral colleagues but were equally likely to have non-PI faculty review their materials. Fewer than 15% of survey respondents, regardless of Programme participation, asked graduate students, their postdoctoral association or career centre or other individuals to review their materials.

Interestingly, the majority of survey respondents (greater than 80%) were employed at institutions with offices for professional development, but many respondents were either unsure (25–30%) if these offices offered editing services or reported these offices did not offer those services (approx. 35–45%; see electronic supplementary material). Of those respondents with professional development offices that offered editing services, the participants (22%) were slightly more likely to have used those services than non-participants (15%; figure 4*b*). Altogether, over 50% of both participants and non-participants indicated either a willingness to use editing services provided by offices of professional development or a desire for those services.

### (e) High satisfaction and improved confidence among Programme participants

**Figure 5.**
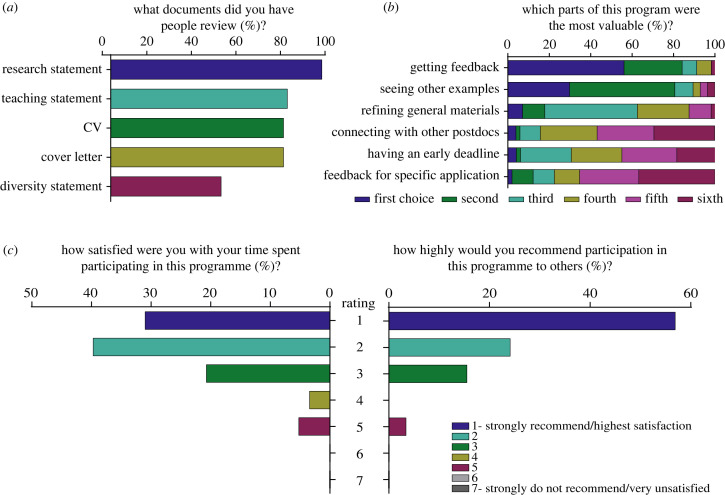
Participants were asked to indicate: (*a*) the documents they had peer-reviewed in the Programme and (*b*) how many people reviewed the documents for each participant and how many people each participant reviewed documents for. (*c*) Participants indicated how satisfied they were with their participation in the FuturePI Reviewing Groups Programme and how highly they would recommend participation in the Programme to others.

Overall, we found that participants in our peer review Programme reported high levels of satisfaction with their experience, seeing example materials from others to be an extremely valuable benefit of participating, and that participating increased confidence in their job application materials. The research statement was the most reviewed document (figure 5*a*) followed closely by the teaching statement, CV and cover letter. The least reviewed document was the diversity statement, which may reflect that fewer job applications require this document or the more personal nature of this particular document. The majority (approx. 65%) of participants reviewed documents and had their documents reviewed by one to five people (figure 5*b*), implying they participated for one to two weeks. Receiving general feedback and seeing other job application examples were reported as the most valuable aspects of participation by over 80% of the participants (see electronic supplementary material). Refining materials, connecting with other postdocs and having an early deadline were rated as comparably less important or valuable aspects of participating in the Programme. Comments on the content were reported as most valuable and copy-editing being the least valuable type of feedback (see electronic supplementary material). Participants reported extremely high satisfaction with their participation in the Programme, with only 8.6% (5/58) giving a rating lower than 5 out of 7 (figure 5*c*). Similarly, 96.5% of respondents (56/58) reported they would be likely or highly likely to recommend participation in the Programme to other colleagues.

When asked about their motivations for participating in the Programme, the respondents were primarily motivated (i) to seek feedback beyond their own laboratory (*n* = 34), especially from peers (*n* = 12) and people outside of their subdiscipline (*n* = 16) and (ii) to use examples from others to better organize their job documents (*n* = 10; electronic supplementary material, figure S1a):

I wanted a broader perspective than could be provided from just the members of my lab, since a faculty search committee will be composed of many people who aren't experts in my area. I also wanted as many eyes as possible to read my documents. And I wanted to see the documents of others to see if anyone had any interesting ideas I could use.

The majority of participants noted, as shown in the representative quote below, that their participation in the Programme improved their confidence in their job application materials, the application process (*n* = 13), and the quality of their materials (*n* = 17), and that they felt more supported during the job application process, which is often stressful and isolating (*n* = 14; electronic supplementary material, figure S1b):

It gave me more confidence, both because someone had looked at my stuff and because I felt like a part of a community and that I didn't have to do this all on my own – there were other great people like me going through the same thing.

By contrast, only three participants felt that their participation had little or no impact.

When asked to provide examples of helpful feedback, the participants noted they received helpful feedback about the grammar and structure of their documents (*n* = 9) and identifying areas needing more clarity (*n* = 8; electronic supplementary material, figure S1c):

Pointing out sections that would be unclear to a broader audience, suggestions on how to highlight future plans (i.e. what will your lab look like/do).

Someone told me ‘This is really cool and I can't wait to see what happens when you get a job!’ which was so nice and affirming to hear – this whole process is so demoralizing and kind of dehumanizing, and it's really hard to get feedback (and praise, honestly) from people who don't already know you. This person offered helpful constructively critical feedback too, but that was such a nice boost.

The majority of participants, 29 out of 42 (69%), had no concerns about participating (electronic supplementary material, figure S1d). When the participants expressed concern, their concerns revolved primarily around competition and plagiarism (*n* = 8) or the lack of relevant feedback from reviewers (*n* = 7):

Some of the feedback I received seemed rushed and as though the other participant hadn't put much effort into reviewing my documents.

I was worried that my reviewers [sic] fields would be very different from my own.

Among the non-participants, 22 out of 38 were aware of the Programme's existence before the survey (electronic supplementary material, figure S2a), but many of these respondents either were not on the job market in the prior year (*n* = 6), were too busy (*n* = 6) or had obtained feedback elsewhere (*n* = 8). Of those that planned to go on the job market soon, most (11/19) planned to participate in the Programme in the future or were undecided (6/19)—very few non-participants (2/19) planned never to participate in the Programme (electronic supplementary material, figure S2c). In our open-ended question about their reasons for not participating, non-participants reported that they generally heard about the Programme too late or did not have job materials ready (*n* = 9), or they wanted feedback from different people than they thought participated (*n* = 6):

The main reason I did not think feedback from the peer reviews would be useful was that I assumed the majority of reviewers were peers who had yet to successfully get a job, and therefore their comments would be less useful than those of people who had successfully applied for and gotten an academic position, or people who had served on search committees. I do not know how this issue could be addressed within the Future PI Slack community, except to ask those who have since moved on to the next career stage to contribute back.

## 4. Discussion

Since 2018, we have been organizing a growing and successful peer-reviewing Programme within FuturePI Slack for faculty job seekers. We consider the Programme a success because of its continual growth and because the vast majority of participants indicated the Programme was helpful and would recommend participation to others in the future. The purpose of this Programme is not to place postdocs into faculty positions, but rather to provide early career researchers with opportunities for peer review to improve their faculty job application materials and for moral and social support during this extremely challenging process. Our survey data indicate that this Programme emphatically accomplished its goals.

Our participants are diverse in their identities, geographical range, fields, current/training institutional type and dream job type, a likely benefit of online organizing which does not limit us to recruiting participants from a specific location or institution. The results of the survey suggest that the Programme benefits from the participants' shared and unique characteristics and experiences both as a method for improving job application materials and as a significant mechanism of peer support and mentorship. The survey data and personal communication with the organizers suggest that the participants found the Programme to be overwhelmingly positive and would highly recommend participation in it to others during their job search. This support is critical given the extremely stressful and frustrating nature of searching for a faculty job [6], especially so for individuals from underrepresented groups like women, people of colour and LGBTQ+ individuals.

Unfortunately, we are unable to comment on the ability of the Programme to improve the likelihood of successfully obtaining a faculty position for two main reasons. First, given the timing of our survey (prior to most hires) and the difficulty in tracking previous participants, we have no data on the outcomes of participant application journeys. Thus, we have no way of testing if participants are more or less likely to achieve a faculty position than non-participants, assuming they had equal probability prior to participation in our Programme. Second, given the differences between the demographics of our pool compared to the general postdoctoral pool, it is more likely that our Programme equalized chances of obtaining a faculty job for our participants compared to the general pool by providing both tangible and intangible support to disadvantaged job applicants (i.e. candidates that lacked supervisor support or a network within their chosen field, candidates from lower-income countries or underrepresented and marginalized populations).

Previous studies have shown that peer review as a form of mentoring and support may be a critical mechanism for increasing the retention and success of early career academics. Peer review offers not only mentoring and professional development but peer support in general also offers unique psycho-social benefits of emotional support from colleagues with shared experience and/or career stage [46,56]. This unique benefit is particularly important for individuals from marginalized or underrepresented groups in academia [31,47,57,58]. Being a postdoc can be much more isolating compared to being a PhD student [35] due to the lack of a cohesive cohort and fewer individuals at the same career level per laboratory. In fact, postdocs are the least likely to feel a sense of belonging across all stages of the scientific journey, from early graduate students to full professors, a feeling that is exacerbated for individuals from underrepresented or marginalized populations [59]. Data suggest, however, that having a scientific and social community plays a significant role in individual success [58,60]. Further, socio-emotional support and encouragement promote persistence in research careers for early career researchers while lack of these types of support is associated with disengagement from research and possibly attrition [61–63]. Social support can even combat, to a small degree, the negative psychological impact of sexism and racism within academia [30].

Our survey data suggest that at least the Programme, and likely FuturePI Slack more broadly, provide vital support to individuals facing greater difficulty in finding mentors for career support, because of either their career trajectory or identity. First, the respondents of this study are more likely to identify as members of historically underrepresented or marginalized groups, e.g. as female (although survey respondents overall are more likely to identify as female [64,65]) or as non-white, than the pool of postdocs in the USA. This result is particularly notable given that, in the life and biomedical sciences at least, men and women are equally represented in early career stages of PhDs and postdocs [17], but women are significantly less likely than men to transition to an independent PI position [15], representing only 40% of assistant professors and 30% of associate professors [14]. The transition from postdoc to independent PI is a major barrier for individuals from underrepresented minority populations [16,18,66]. However, targeted interventions (e.g. peer review programmes or workshops) can significantly increase postdoc confidence in their ability to apply to faculty jobs, which is predicted to increase persistence [31] at the career stage when these individuals are most likely to ‘leak’ out of the pipeline. The demographics of our survey respondents likely also influenced the broad range of jobs our participants apply to; women and underrepresented minority postdocs are more likely to be interested in pursuing both research-intensive and teaching-intensive jobs, rather than only research-intensive jobs, and postdocs who are three or more years into their position are less committed to remaining in academia than postdocs in their first or second year [61]. Alternatively, postdocs who receive less mentoring from their primary supervisor are less likely to pursue an academic research career [34].

Further, participants were less likely to have their PI or fellow postdocs review their job application materials than non-participants. This difference may be due to a lack of support more broadly and/or because nearly 40% of our respondents switched fields between their PhD and their current position, a trend that is becoming increasingly common [67]. As a result, these postdocs may lack a broad network within their current field. Compared to non-participant respondents, the participants have spent more years on the job market, applied to more jobs and a wider range of jobs, possibly reflecting a wider net cast by individuals who have been searching for a position for longer. Lastly, the responses to the open-ended survey questions on the benefits of participating in the Programme confirmed that participants frequently received positive affirmation on the quality of their materials, which helped them to gain confidence and combat imposter syndrome. Multiple respondents, in commenting on the difficulty and stress of searching for a faculty position, specifically mentioned that participating in the Programme helped them to feel less alone (e.g. [56,68]) and previous surveys have reported that postdocs found the process of applying for jobs to be easier when they had a strong network of support [13]. The participants were further emotionally bolstered by the act of helping others to improve their materials and the idea that as a community, early career academics all rise by helping each other. Community building as a career development strategy provides opportunities [57], especially for historically excluded groups, to build social capital and networks that may enhance their career development [69]. Similar peer review programmes could fulfil the same function by providing peer support and mentorship for early career academics at their institutions, particularly for those who may lack that support elsewhere.

In addition to quantifying the utility of the Programme, another goal of this study was to identify areas of potential improvement and best practices for participating by combining both the survey and additional participant feedback sent directly to the organizers. For example, in 2022, we began recruiting participants for the Programme in June and organized the first week of peer-reviewing in July instead of waiting until August. Because the COVID-19 pandemic has highlighted the need for flexibility, we also transitioned from sending out materials on Monday and asking for feedback by Friday to asking that materials be sent out on Thursday with feedback due back on Monday. One challenge we have consistently encountered is ensuring full participation for and from everyone who signs up. Often, potential participants sign up immediately after the Programme announcement on FuturePI Slack, but weeks later, these individuals may find themselves with too much other work to participate. We now send an email the day before to everyone who has signed up requesting confirmation that they are still willing and able to participate before assigning groups. Even with this precaution, however, a few times a year even confirmed participants discover they are unable to participate. In these instances, we try to maintain flexibility for those participants and ensure the full benefits of participation for the remaining group members. We now ask participants to notify us if a group member failed to send out their materials or failed to send back feedback on the documents of their group members. If a participant fails to do either of these actions more than twice after confirming their desire to participate, they are removed from participation for the rest of the year in order to ensure other participants do not miss out on feedback from group members too often. So far, we have not had to remove a participant. We also ask to be notified of any unprofessional review comments and immediately bar individuals who provide such comments from participating in any future rounds of peer review [70–72]. Lastly, participants have communicated anecdotally to the organizers their struggle to find peers with experience outside of the US system to review materials for non-US jobs, which may differ in the documents required and their structure. We hope that as the FuturePI Slack group continues to grow and gain new members, a more globally diverse pool of peer reviewers will be available. Furthermore, as the Programme grows, organizers could recruit peer reviewers from previous cycles who have specific experiences that match participant job goals. Multiple participants suggested the following best practices guidelines, which we plan to include as planning advice for future Programme participants and for organizations that wish to launch their own version: (i) participate early when documents are still quite rough in order to get ‘big picture’ or overall feedback on the ideas and organization and (ii) participate more than once to get a breadth of feedback from at least four to six peer reviewers, but on a timeline of every other week to allow for significant revision of early drafts.

Finally, the last goal of this study was to provide a model for other organizations wishing to develop similar peer-reviewing programmes by providing a detailed description of our organizing methodology (see Programme description) as well as evidence that such programmes are needed and beneficial, in part because trainees' perceived institutional support drives career search efficacy for postdocs [73]. Already, the authors have received anecdotal reports from former participants organically replicating the goals and structure of this Programme at other organizations (e.g. Plant Postdoc Slack and Victoria University of Wellington Postdoctoral Society) for graduate students, postdocs and other early career academics. As indicated in our survey, despite the ubiquity of offices for professional development whose services might be of use to them, most survey respondents were either unsure if editing services were offered or chose not to take advantage of these services, indicating a need for more specialized programmes or more targeted advertisements by these offices. Moreover, the organization of similar peer-reviewing programmes is not limited to universities and colleges. Discipline-specific professional societies and organizations or broader national organizations like the National Postdoctoral Association in the USA could organize similar programmes, either as a workshop at annual conferences or a multi-week programme over the longer term. In fact, the sponsoring of such groups by professional scientific societies has been spontaneously suggested by postdocs in surveys as a way to improve their support of postdocs [8]. Similar programmes organized at a localized level could also provide support for dealing with the variability in expectations for job applications across the globe.

To lower the effort required to start a similar programme, we include a ‘starter’ package of materials we routinely use in the electronic supplementary material. If societies or institutions are interested in implementing their own programme, we suggest first emailing the pool of potential participants with a short survey to gauge the level of interest in participating, as well as the best options for programme structure. For example, the initial survey could ask (i) what documents participants might want reviewed, as this differs by institution type and geographical location, (ii) when during the calendar year such a programme might be best offered as the timing of job applications may differ across the globe, (iii) how many cycles of peer review potential participants might wish to participate in and (iv) how much time participants might need to peer review materials. Organizers might also use this survey to ask about the necessity for offering workshops or information sessions on the process of applying for faculty jobs and the parts of a faculty job application, or if institutions or societies already offer such workshops, these can serve as the springboard for a peer review programme. Once this baseline information is collected, organizers can adapt the organizational structure and materials included in this paper to their specific needs and structure. Furthermore, the focus of such a programme need not be limited to job application materials—it can also be expanded to offer peer review for the other aspects of the faculty application journey such as job talks or chalk talks (e.g. [74]) or shifted to other types of academic documents like grant applications, course syllabi or reappointment/tenure dossiers.

Ultimately, programmes like the FuturePI Reviewing Groups Programme provide an opportunity to improve the quality of one's job application materials, which may, in turn, improve one's odds of success in attaining an independent faculty position. However, programmes that build peer support and mentorship networks for early career academics may also play a role in retaining and strengthening a diverse academic workforce despite the structural leaks in the pipeline. Our hope is that this Programme description inspires other organizations to create similar programmes to support vulnerable early career academics in their search for independence.

## Data Availability

Survey questions are included in the electronic supplementary material as are anonymized demographic data from survey respondents and open-ended survey responses. The data are provided in electronic supplementary material [75].
